# Effects of Short-Term Nutritional Interventions on Right Ventricular Function in Healthy Men

**DOI:** 10.1371/journal.pone.0076406

**Published:** 2013-09-23

**Authors:** Ralph L. Widya, Sebastiaan Hammer, Mariëtte R. Boon, Rutger W. van der Meer, Johannes W. A. Smit, Albert de Roos, Patrick C. N. Rensen, Hildo J. Lamb

**Affiliations:** 1 Department of Radiology, Leiden University Medical Center, Leiden, the Netherlands; 2 Department of Endocrinology and Metabolism, Leiden University Medical Center, Leiden, the Netherlands; Centre Hospitalier Universitaire Vaudois, Switzerland

## Abstract

**Background:**

A physiological model of increased plasma nonesterified fatty acid (NEFA) levels result in myocardial triglyceride (TG) accumulation, which is related to cardiac dysfunction. A pathophysiological model of increased plasma NEFA levels result in hepatic steatosis, which has been linked to abnormal myocardial energy metabolism. Hepatic steatosis is accompanied by hepatic inflammation, reflected by plasma cholesteryl ester transfer protein (CETP) levels. The current study aimed to investigate effects of these models via different nutritional interventions on right ventricular (RV) function.

**Methods:**

Fifteen men (age 25.0±6.6 years) were included and underwent magnetic resonance imaging and spectroscopy in this prospective crossover intervention study. RV function, myocardial and hepatic TG content, and CETP levels were assessed on three occasions: after normal diet, very low-calorie diet (VLCD, physiological model) and high-fat high-energy (HFHE, pathophysiological model) diet (all 3-days diets, randomly ordered, washout phase at least 14 days).

**Results:**

VLCD induced a decrease in mean E deceleration by 27%. Myocardial TG content increased by 55%, whereas hepatic TG content decreased by 32%. Plasma CETP levels decreased by 14% (all P<0.05). HFHE diet induced a decrease in E/A by 19% (P<0.05). Myocardial TG content did not change, whereas hepatic TG content increased by 112% (P<0.01). Plasma CETP levels increased by 14% (P<0.05).

**Conclusions:**

These findings show that RV diastolic function is impaired after short-term VLCD and HFHE diet in healthy men, respectively a physiological and a pathophysiological model of increased plasma NEFA levels. After short-term VLCD, myocardial lipotoxicity may be of importance in decreased RV diastolic function. RV diastolic dysfunction is accompanied by increased hepatic TG content and plasma CETP levels after short-term HFHE diet, suggesting that systemic inflammation reflecting local macrophage infiltration in the heart may be involved in RV dysfunction.

## Introduction

Nutritional interventions have shown to be useful for studying plasma nonesterified fatty acid (NEFA) levels and plasma triglyceride (TG) levels, and the flexibility of myocardial TG content and its relation to myocardial function [1–4]. Prior dietary intervention studies have focused on LV function. The effects of nutritional interventions on right ventricular (RV) function have not yet been studied. RV function is important in patient risk stratification in heart failure [5,6] and prediction of developing atrial fibrillation [7]. Advanced RV dysfunction and fibrosis are associated with exercise limitation, lethal ventricular arrhythmias, sudden death, and reduced RV cardiac output [8,9]. Moreover, it has been shown that RV dimensions and function are impaired in type 2 diabetes [10,11], a disease with abnormal fuel flux and energy imbalance as metabolic hallmarks.

It has been reported that short-term exposure to a very low-calorie diet (VLCD) increases plasma NEFA levels [12]. Excessive plasma NEFA levels result in myocardial TG accumulation in animal models of obesity and type 2 diabetes [13,14]. In such models, TG accumulation in cardiomyocytes is related to cardiac dysfunction [15–17] and an increased susceptibility for cardiac ischemia [18]. Complex mechanisms, most likely involving intermediates of NEFA metabolism and oxidative stress [13,17,19], are responsible for this phenomenon called “myocardial lipotoxicity”. It has previously been shown that a VLCD induces an increase in myocardial TG content and a concomitant decrease in left ventricular (LV) diastolic function in lean healthy men [2]. These observations suggest that when NEFAs are taken up in excess of fatty acid oxidation, myocardial TG content increases. We therefore hypothesized that such a physiological model, induced by short-term exposure to a VLCD, would also impair RV function in lean healthy men.

Increased NEFA levels induced by a short-term high-fat high-energy (HFHE) diet caused increased hepatic TG content, but did not influence myocardial TG content or LV function in the same healthy population [3]. Hepatic steatosis has been linked to abnormal myocardial energy metabolism [20,21]. In addition, hepatic steatosis may be accompanied by hepatic inflammation. The liver macrophage is suggested to be a primary contributor to plasma cholesteryl ester transfer protein (CETP) mass [22]. Plasma CETP may therefore serve as a biomarker for the hepatic macrophage content and, thus, hepatic inflammation. Interestingly, short-term (5 days) exposure to HFHE diet increases plasma CETP along with an increase in macrophage expression markers in muscle biopsies, indicating that HFHE diet causes macrophage recruitment to tissues, including liver and muscle but possibly also the heart (MR Boon, MSc, and PCN Rensen, PhD, unpublished data, 2012). Activated macrophages in the myocardium contribute to cardiac myocyte contractile dysfunction [23]. We, therefore, hypothesized that in a pathophysiological model created by a short-term HFHE diet systemic macrophage recruitment (as reflected by increased plasma CETP levels) is linked to both hepatic tissue changes and RV dysfunction in lean healthy men.

Therefore, the aim of this study was to investigate the effects on right ventricular function in lean healthy men in two different conditions: the first was a physiological model created by a short-term caloric restriction; the second was a pathophysiological model created by a short-term high-fat diet. 

## Methods

### Ethics Statement

This study was approved by the Medical Ethical Committee of the Leiden University Medical Center and was conducted in accordance with the Declaration of Helsinki. Written informed consent was obtained from all participants.

### Subjects

Fifteen healthy men were included in this prospective crossover intervention study. Inclusion criteria were: 1. age > 18 years; 2. no known acute or chronic disease based on history, physical examination, or standard laboratory tests (blood counts, serum creatinine, alanine aminotransferase, aspartate aminotransferase, and electrocardiogram). Exclusion criteria were drug treatment, smoking, substance abuse, hypertension, or impaired glucose tolerance (as confirmed by a 75-g oral glucose intolerance test). All subjects performed exercise (walking, running, biking) regularly (3-5 hours weekly), but none engaged in high-performance sports. Part of these data was previously published in reports describing hepatic and myocardial TG accumulation [2,3]. To study effects of a HFHE diet on RV function similar volunteers were used [3]. To study the effects of a VLCD, one volunteer was added [2].

### Study design

**Figure 1:**
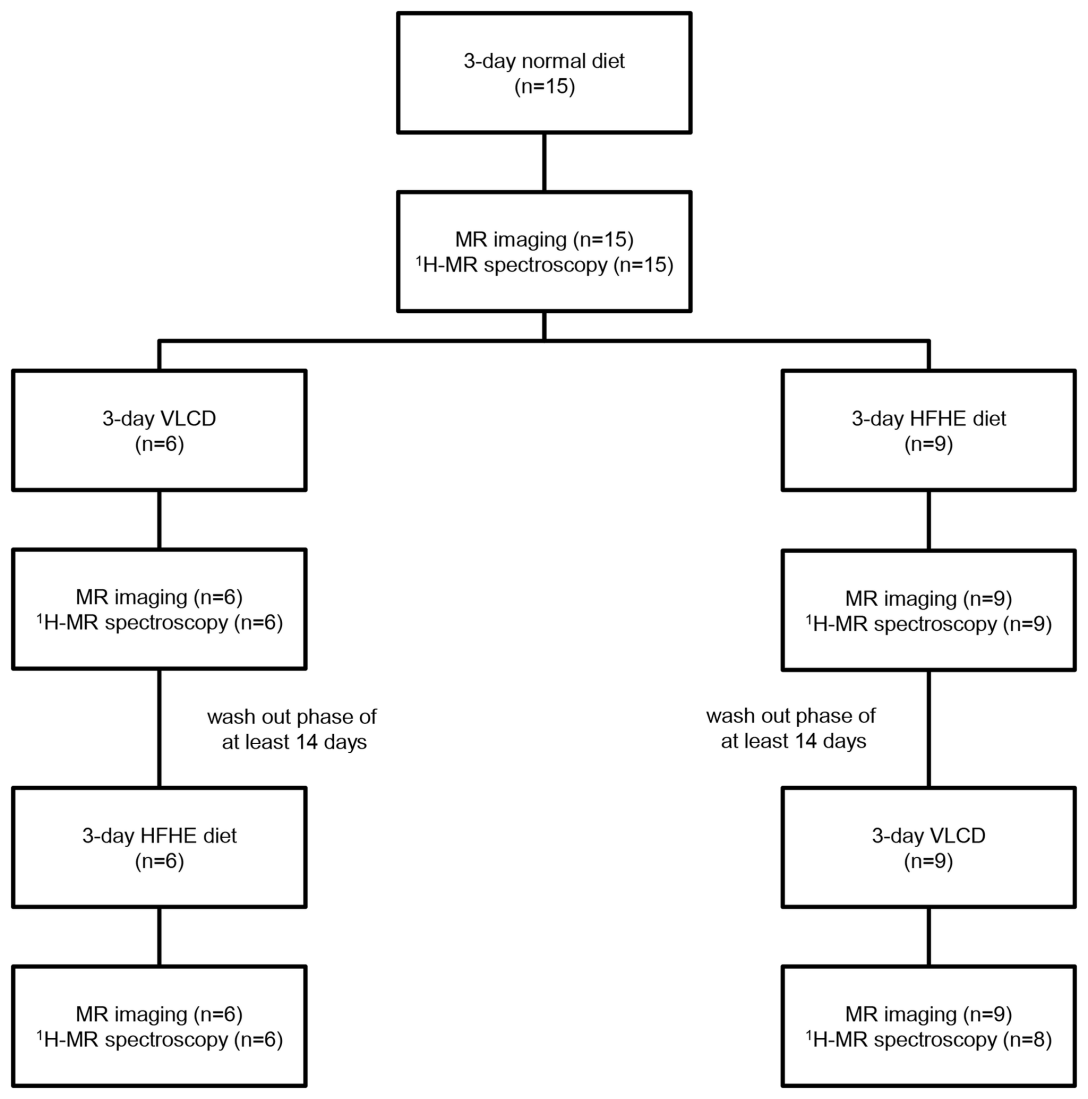
Workflow of the study. Baseline data were collected after a 3-day normal diet. Next, subjects underwent a 3-day very low-calorie diet (VLCD) and a 3-day high-fat high-energy (HFHE) diet. The sequence of the nutritional interventions was randomly assigned to minimize influences caused by the sequence of the interventions. Both interventions were separated by a washout phase of at least 14 days.

All subjects underwent venous blood sampling and magnetic resonance (MR) scanning in the afternoon at three different occasions. Before every visit, subjects were instructed to follow different dietary regimes. First, baseline data were collected after a 3-day normal diet, which consisted of approximately 2100 kcal/day. Next, subjects underwent a 3-day VLCD and a 3-day HFHE diet. The sequence of the nutritional interventions was randomly assigned to minimize influences caused by the sequence of the interventions. Both interventions were separated by a washout phase of at least 14 days (Figure **1**). The VLCD consisted of 471 kcal, 50.2 g carbohydrates, and 6.9 g fat, of which 0.94 g saturated fat (Modifast Intensive, Nutrition & Santé Benelux, Breda, the Netherlands) per day. The intake of the HFHE diet was similar to the reference diet, complemented with 800 mL cream every day. The cream added 2632 kcal/day, totaling approximately 4732 kcal/day. Subjects were instructed to maintain a sufficient fluid intake (at least 1.5 L daily). Alcohol use was not allowed during the diets. Every meal was consumed 4 h prior to data collection.

### Proton MR spectroscopy

Myocardial and hepatic TG content were assessed by proton MR spectroscopy as described previously [2,3,24]. In short, MR spectroscopy studies were performed using a 1.5-T whole-body MR scanner (Gyroscan ACS/NT15; Philips, Best, The Netherlands) with subjects in the supine position at rest. Cardiac proton MR spectra were obtained from the interventricular septum using a point-resolved spectroscopy sequence to acquire single voxel MR spectroscopic data from an 8-mL voxel. Spectra were acquired at end systole, with an echo time (TE) of 26 ms and a repetition time (TR) of at least 3000 ms. A total of 1024 data points was collected using a 1000-Hz spectral width and averaged over 128 acquisitions. The spectroscopic data acquisition was ECG triggered, and respiratory gating based on navigator echoes was applied to minimize breathing influences [24]. Without changing any parameter, spectra without water suppression with a TR of 10 s and four averages were obtained, to be used as an internal standard.

Proton MR spectroscopy of the liver was performed with an 8-mL voxel positioned in the liver, avoiding gross vascular structures and adipose tissue depots. The 12^th^ thoracic vertebra was used as a landmark to ensure the same position of the voxel during both visits. Spectra were obtained without respiratory motion compensation using similar parameters as described previously. Only 64 averages were collected with water suppression.

All spectroscopic data were fitted using Java-based MR user interface software (jMRUI version 2.2; developed by A. van den Boogaart, Katholieke Universiteit Leuven, Leuven, Belgium) [25] as described before [24]. The percentage of myocardial and hepatic TG signals relative to the water signal was calculated as: (signal amplitude of TGs) / (signal amplitude of water) x 100.

### MR imaging

Velocity-encoded MR imaging is a noninvasive imaging modality extensively used for blood flow assessment [26]. Moreover, cardiovascular MR imaging has become the reference standard for the assessment of RV function and volumes, yielding high accuracy and good reproducibility [27,28]. MR scanning was performed on a 1.5-T whole body MRI scanner (Gyroscan ACS/NT15; Philips, Best, the Netherlands), with subjects in the supine position at rest as previously described [2,3]. In short, RV dimensions and systolic function were assessed by imaging the entire heart in short-axis orientation using electrocardiographically gated breath-hold balanced steady-state free precession imaging. Endocardial contours of the RV were manually drawn in end-diastolic phase and end-systolic phase as previously described [11]. Imaging parameters were as follows: repetition time (TR)=3.3 ms, echo time (TE)=1.67 ms, flip-angle=35°, slice thickness=10 mm, slice gap=0 mm, field of view=400 x 400 mm, reconstructed matrix size=256x 256. Dimensions were indexed to body surface area, resulting in end diastolic volume index (EDVI) and end systolic volume index (ESVI).

Body surface area was calculated according to the Mosteller formula: (weight (kg) × height (cm)/3600)^0.5^ [29].

RV diastolic function was determined by measuring blood flow across the tricuspid valve. Diastolic parameters included peak filling rates of the early filling phase (E) and atrial contraction (A), and their ratio (E/A). Also, the peak and mean deceleration gradient of E was calculated. Electrocardiographically gated gradient echo sequences with velocity encoding were performed. The following imaging parameters were used: TR=6.5 ms, TE=1 ms, flip-angle=20°, slice thickness=8 mm, field of view=350 x 350 mm, matrix size=256x 256, EPI-factor=3, velocity encoding gradient=100 cm/s. Image postprocessing was performed with in-house-developed software packages (MASS and FLOW, Medis, Leiden, the Netherlands).

### CETP

Plasma samples were obtained at all three occasions, stored in aliquots at -80°C, and analyzed after thawing once in a single laboratory (Dept. Endocrinology, Leiden, the Netherlands). Plasma CETP concentration was quantified using kit CETP ELISA Daiichi (Daiichi Pure Chemicals, Tokyo, Japan).

### Statistical analysis

Statistical analyses were performed using SPSS Statistics version 20 (IBM, Armonk, NY). Data are expressed as means ± SD. The study conditions were compared by a two-tailed paired t test. Because a change in heart rate influences the interpretation of RV functional measures, a linear mixed model was performed to correct for heart rate when altered after a diet. Finally, multivariate linear regression models were used to study correlations of differences of myocardial TG content with differences of RV diastolic function after VLCD, and differences of hepatic TG content and CETP with differences of RV diastolic function after HFHE diet. Because E/A decreases with age, we adjusted for age in the multivariate linear regression models [30]. Furthermore, we also adjusted for the difference in heart rate in the condition after HFHE diet. A P-value <0.05 was considered statistically significant. 

## Results

Part of these data, including MR spectroscopy data, was previously published [2,3]. Mean age of the studied subjects was 25.0±6.6 years.

### Very Low-Calorie Diet

Participant characteristics at baseline and after the VLCD are shown in Table **1**. BMI range at baseline was 19.4-28.2 kg/m^2^ and after VLCD 19.2-27.4 kg/m^2^. BMI decreased from 23.4±2.5 to 23.1±2.4 kg/m^2^ and body surface area decreased from 2.10 ± 0.21 to 2.08 ± 0.21 m^2^ (both P<0.01). Plasma glucose levels decreased from 4.9±0.3 to 4.3±0.4 mmol/L, and plasma NEFA levels increased from 0.54±0.29 to 1.12±0.38 mmol/L (both P<0.001).

**Table 1:** Participant characteristics at baseline and after short-term caloric restriction and short-term high-fat diet.

	**Baseline**	**VLCD**	**HFHE**
Body mass index (kg/m^2^)	23.4 ± 2.5	23.1 ± 2.4*	23.6 ± 2.5
Body surface area (m^2^)	2.10 ± 0.21	2.08 ± 0.21*	2.11 ± 0.21
Systolic blood pressure (mmHg)	123 ± 13	118 ± 10	125 ± 13
Diastolic blood pressure (mmHg)	67 ±8	62 ± 8*	64 ± 8
Heart rate (bpm)	60 ± 9	61 ±10	69 ± 11*
Plasma glucose (mmol/L)	4.9 ± 0.3	4.3 ± 0.4*	5.0 ± 0.4
Plasma insulin (mU/L)	9.1 ± 4.6	7.7 ± 4.3	21.4 ± 8.8*
Plasma triglycerides (mmol/L)	1.3 ± 0.4	0.9 ± 0.3*	2.9 ± 1.1*
Plasma NEFA (mmol/L)	0.54 ± 0.29	1.12 ± 0.38*	0.92 ± 0.33*
Total cholesterol (mmol/L)	4.7 ± 1.2	4.7 ± 1.3	5.0 ± 1.3
HDL cholesterol (mmol/L)	1.5 ± 0.4	1.5 ± 0.4	1.6 ± 0.4*

Values are means ± SD. HDL = high-density lipoprotein. HFHE = high-fat high-energy. NEFA = nonesterified fatty acids. VLCD = very low-calorie diet. *P<0.05 compared to baseline. These data are partly based on previous reports [2,3] (see Methods section).

MR imaging results are depicted in Table **2**. RV dimensions changed, as shown by a decreased EDV and ESV (251 ± 39 to 238 ± 39 mL and 131 ± 22 to 123 ± 23 mL, respectively). After indexing for body surface area, RV EDVI decreased from 119.5 ± 12.6 to 114.4 ± 14.0 mL/m^2^ (P<0.05), while a decrease in RV ESVI did not reach significance (P=0.056). Systolic function did not change. The mean deceleration of the early diastolic flow across the tricuspid valve decreased from 1.81±0.49 to 1.33±0.32 mL/s^2^ x 10^-3^ (P<0.01).

**Table 2:** RV parameters at baseline and after short-term caloric restriction and short-term high-fat diet.

	**Baseline**	**VLCD**	**HFHE**
**Dimensions**			
End diastolic volume index (mL/m^2^)	119.5 ± 12.6	114.4 ± 14.0*	118.1 ± 13.2
End systolic volume index (mL/m^2^)	62.1 ± 6.9	58.7 ± 8.7	59.9 ± 9.4
**Systolic function**			
Stroke volume (mL)	121 ± 21	116 ± 18	123 ± 19
Cardiac output (L/min)	7.5 ± 1.7	7.2 ± 1.1	8.5 ± 1.1
Ejection fraction (%)	48.0 ± 3.1	48.7 ± 2.8	49.4 ± 4.0
**Diastolic function**			
E peak flow rate (mL/s)	422 ± 92	391 ± 79	400 ± 70
Peak E deceleration (mL/s^2^ x 10^-3^)	3.17 ± 1.01	2.63 ± 0.78	2.60 ± 0.97
Mean E deceleration (mL/s^2^ x 10^-3^)	1.81 ± 0.49	1.33 ± 0.32*	1.40 ± 0.49
A peak flow rate (mL/s)	301 ± 84	310 ± 76	355 ± 99
E/A	1.47 ± 0.43	1.30 ± 0.27	1.19 ± 0.29*

Values are means ± SD. A = atrial contraction. E = early filling phase. HFHE = high-fat high-energy. VLCD = very low-calorie diet. *P<0.05 compared to baseline.

**Figure 2:**
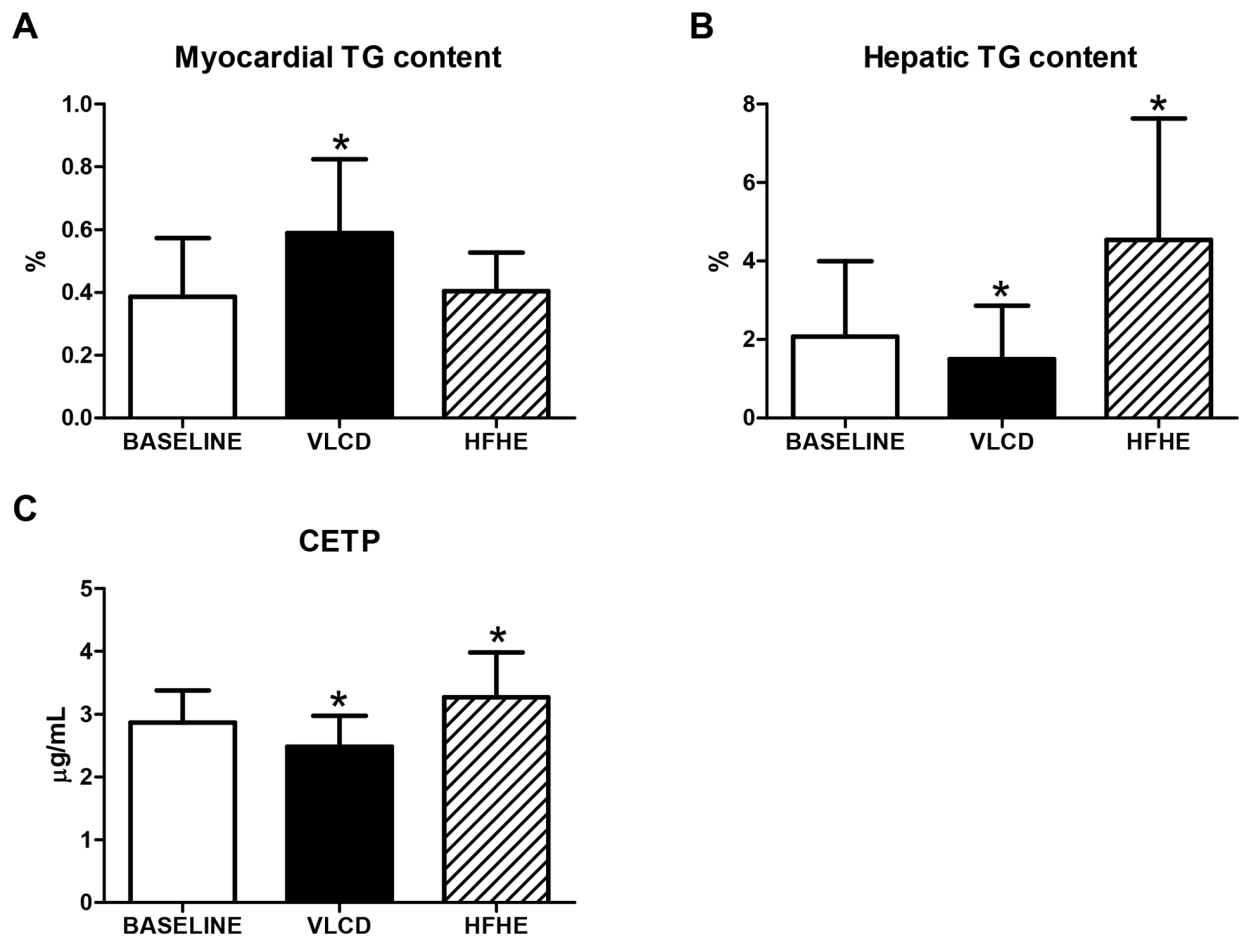
Myocardial and hepatic TG content and plasma CETP levels after short-term caloric restriction and short-term high-fat diet. Myocardial (A) and hepatic (B) triglyceride content and plasma CETP levels (C) at baseline (white bars), after short-term caloric restriction (black bars), and after short-term high-energy diet (hatched bars). CETP = cholesteryl ester transfer protein. HFHE = high-fat high-energy. TG = triglyceride. VLCD = very low-calorie diet. * P<0.05. These data are partly based on previous reports [2,3] (see Methods section).

MR spectroscopy failed in one subject for unknown reasons leading to insufficient spectral resolution. As a result 14 men were included in the myocardial and hepatic fat analyses. Myocardial TG content increased by 55% from 0.38±0.19 to 0.59±0.24% (P<0.01) (Figure **2A**), whereas hepatic TG content decreased by 32% from 2.19±1.94 to 1.50±1.36% (P<0.05) (Figure **2B**). Plasma CETP levels decreased by 14% from 2.87±0.51 to 2.48±0.49 µg/mL (P<0.01) (Figure **2C**), while HDL-cholesterol was not changed (Table **1**).

No correlations were found between the difference in myocardial TG content and the difference in any of the RV diastolic function parameter.

### High-Fat High-Energy diet

Participant characteristics at baseline and after the HFHE diet are depicted in Table **1**. BMI range after the HFHE was 19.9-27.9 kg/m^2^. BMI and body surface area remained similar, as well as plasma glucose levels. Plasma NEFA levels increased from 0.54±0.29 to 0.92±0.33 mmol/L (P<0.01). The 3-day HFHE diet (hereafter, high-fat diet) increased heart rate from 60±9 to 69±11 bpm (P<0.01). For this reason changes in RV dimensions and function were corrected for heart rate, corrected data are shown.

RV dimensions remained similar. Systolic function did not change. E/A was decreased from 1.47±0.43 to 1.19±0.29 (P<0.05) (Table **2**).

Myocardial TG content did not change (Figure **2A**), whereas hepatic TG content increased by 112% from 2.01±1.79 to 4.26±2.78% (P<0.01) (Figure **2B**). Plasma CETP levels increased by 14% from 2.87±0.51 to 3.27±0.72 µg/mL (P<0.05) (Figure **2C**). HDL-cholesterol increased from 1.5±0.4 to 1.6±0.4 mmol/L (P<0.05) (Table **1**).

A correlation was found between the increase in hepatic TG content and decrease in E/A (β -0.099, R^2^ 0.364, P=0.05). The increase of plasma CETP levels did not correlate with the difference in any of the RV diastolic function parameters. 

## Discussion

The main finding of the current study is that in the physiological as well as in the pathophysiological model of increased NEFA levels, induced by respectively short-term caloric restriction and short-term high fat diet, RV function is decreased in healthy men. Furthermore, plasma CETP levels decrease after short-term caloric restriction and increase after short-term high-fat diet.

### Effects after Very Low-Calorie Diet

It has been recognized that short-term caloric restriction causes tissue-specific partitioning of plasma TG and/or fatty acids among non-adipose organs in healthy subjects, with respect to the liver and the heart. In a prior study, short-term caloric restriction induced an increase of myocardial TG, whereas hepatic TG content decreased. Furthermore, LV diastolic function was decreased [2]. We hypothesized that such a physiological model would also impair RV function. To the best of our knowledge, this is the first study to show decreased RV diastolic function after VLCD in lean healthy men. Possible causes include impaired relaxation through the activation of phospholipases after a short-term caloric restriction causing changes in calcium homeostasis [31–33]. Another explanation for decreased diastolic function might be a relatively higher reliability of the heart on NEFA, since plasma NEFA levels were found to be increased and plasma glucose levels decreased after short-term caloric restriction. Therefore, the myocardium may not benefit from potential favorable effects of carbohydrate oxidation on myocardial function and efficiency [2,34,35].

The mean deceleration of the early diastolic flow across the tricuspid valve was decreased after short-term caloric restriction, similar to the mean deceleration of the early diastolic flow across the mitral valve in the LV [2]. Even though the differences of the other diastolic function parameters after short-term caloric restriction did not reach statistical significance, a trend was observed of a decreased ratio of the peak filling rates of the early filling phase to atrial contraction, indicating a change in relaxation of the right ventricle. Furthermore, RV volumes were decreased after short-term caloric restriction compared to baseline. After indexing for body surface area, RV EDVI was significantly lower. Decreased RV volumes and impaired RV function have also been implicated as components of the diabetic cardiomyopathy phenotype [11].

We performed multivariate linear regression analyses to assess the relationship between the change in myocardial TG content and RV diastolic dysfunction. However, we did not find any correlations. Nonetheless, myocardial TG accumulation may be a marker of many hormonal, metabolic, and biophysical changes within the myocardium that occur during a VLCD, and this complex interplay may be of influence on myocardial diastolic function.

Plasma CETP levels decreased by 14%. This is in full accordance with previous findings of a prolonged caloric restriction which resulted in a marked decrease in hepatic TG content and plasma CETP levels in obese type 2 diabetic patients [36]. CETP is a hydrophobic plasma glycoprotein that is involved in the exchange of cholesteryl esters and TG between HDL and apoB-containing lipoproteins (e.g., VLDL and LDL), resulting in a net transfer of cholesteryl esters from HDL to apoB-containing lipoproteins [37]. Liver macrophages largely contribute to hepatic CETP expression, and attenuation of hepatic steatosis, accompanied by attenuation of liver inflammation, as reflected by reduced macrophage content, by the anti-dyslipidemic drug niacin decreased hepatic CETP expression and the plasma CETP level [22]. To our knowledge, this study is the first to report a decrease in plasma CETP level already after three days of caloric restriction, suggesting that hepatic inflammation (i.e. macrophage content) is reduced.

### Effects after High-Fat High-Energy diet

We created a pathophysiological model of increased plasma NEFA levels via a short-term HFHE diet and hypothesized that RV function would be decreased as a result of hepatic inflammation. Remarkably, RV diastolic function was altered after short-term high-fat diet whereas LV function was previously found to remain similar in the same study group [3]. This discrepancy might be caused by the observed increase of hepatic TG content after short-term high-fat diet leading to reduced compliance of the hepatic parenchyma. Hypertrophied hepatocytes might cause hepatic vein compression and subsequently decrease vein phasicity [38]. The hepatic veins drain blood from the hepatic sinusoids toward the inferior vena cava. As a result of abnormal flow in the hepatic veins, blood flow in the inferior vena cava may also be anomalous. It has been recognized that the hepatic vein doppler waveform alters from triphasic to biphasic or monophasic in patients with non-alcoholic fatty liver disease (NAFLD) [38,39]. The early passive filling of the right atrium and ventricle may be disturbed, causing a decreased E/A. Unfortunately, we did not perform duplex doppler ultrasonography to investigate the waveforms of the hepatic veins.

Alternatively, taken together the rise in plasma CETP levels with our previous finding that short-term high-fat diet increases the expression of macrophage genes in muscle biopsies (MR Boon, MSc, and PCN Rensen, PhD, unpublished data, 2012), it is possible that short-term high-fat diet results in macrophage activation and recruitment into tissues including liver, muscle and possibly also the heart. Activated macrophages that adhere to intercellular adhesion molecule-1 (ICAM-1) expressed on cardiac myocytes contribute to cardiac myocyte contractile dysfunction [23]. Furthermore, macrophages and macrophage-derived mediators have been implicated in myocardial dysfunction in a number of myocardial inflammatory states [23], including ischaemic and non-ischaemic cardiomyopathy [40]. A recent animal study showed that several inflammatory cytokines were upregulated accompanied by macrophage infiltration in lipotoxic cardiomyopathy, and that macrophages contribute to adverse cardiac remodeling in response to lipid overload [41]. It may be hypothesized that RV diastolic dysfunction is an early manifestation of myocardial macrophage infiltration after short-term high-fat diet.

Short-term high-fat diet induced a more than 2-fold increase in hepatic TG content in these subjects, whereas it did not influence myocardial TG content [3]. It has now been recognized that a combined hyperglycemia and hyperinsulinemia induce short-term myocardial TG accumulation and alterations in LV function in normal subjects, indicating that postprandial and/or chronic hyperglycemia and hyperinsulinemia might be involved in myocardial steatosis in metabolic diseases [42]. Healthy subjects showed hyperinsulinemia but no hyperglycemia in response to high-fat diet [3]. This could explain the absent myocardial steatosis and possibly the absence of LV dysfunction [3].

### Limitations

Some potential limitations should be addressed. First, our study sample is small and the study may be underpowered to perform adequate correlative statistical analyses. Therefore, further studies with larger population should be performed. Second, we did not measure flow of the hepatic vein and inferior vena cava that could support the hypothesis that hepatic steatosis may result in anomalous flow in the hepatic vein and the inferior vena cava leading to disturbed filling of the RV. We measured hepatic TG content and plasma CETP levels as markers for hepatic inflammation. However, further studies need to be initiated combining duplex doppler ultrasonography of the hepatic vein and inferior vena cava and assessment of RV function.

In conclusion, RV function is impaired after short-term caloric restriction in healthy men. Furthermore, RV function is also impaired after short-term high-fat diet. Whereas the response of the right ventricle to short-term caloric restriction seems analogous to the left ventricle, it shows a differential response to short-term high-fat diet as compared to the left ventricle. RV dysfunction is accompanied by increased hepatic TG content and plasma CETP levels in short-term high-fat diet, suggesting that systemic inflammation reflecting local macrophage infiltration in the heart may be involved in RV dysfunction.
